# Supplementary material for: Drug repurposing for aging research using model organisms
Source: Aging Cell. 2017 Jun 16;16(5):1006–15. doi: 10.1111/acel.12626 (PMC5595691; doi:10.1111/acel.12626)
Supplement: Supplementary file 7 — Data S1 Zip‐Archive of all report cards. [file ACEL-16-1006-s007.zip › RC_ODH.pdf]

ODH

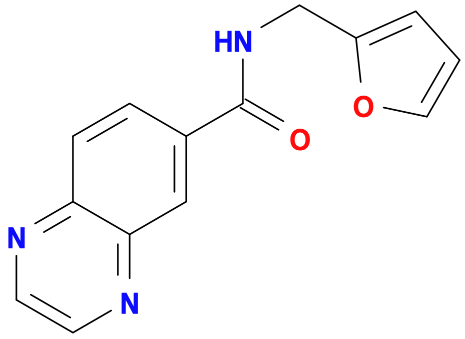

Database identifiers

ChEMBLCompound	CHEMBL1408855
ZINC	ZINC04869926
eMolecules	2269217

Ranking

	Rank	Score
Drosophila	427/697	0.335
C. elegans	378/591	0.156

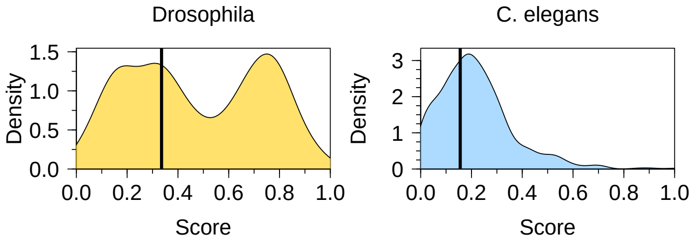

	Ageing implication	Domain conservation	Binding site conservation	Binding affinity	Bioavailability	Lipinski	Promiscuity	Purchasability	Drug approval	**Total**
Drosophila	0.563	0.891	0.912	0.574	(0.9)	0.0	-0.0	0.1	0.0	0.335
C. elegans	0.563	0.821	0.82	0.574	0.258	0.0	-0.0	0.1	0.0	0.156

Names

No synonyms found

Roles

ChEBI entry None has no roles

Status

Approved drug (according to ChEMBL)	No
Number of Rule of 5 violations	0
Binding affinity to original target in log units (RF-Score prediction)	5.3
Burns *C. elegans* bioavailability prediction	-1.72

Compound Target Characteristics

Serine/threonine-protein kinase Chk2

Best gene implication in ageing for this target family came from gene O96017 annotated in UniProt release 2014_02. Annotation GO subterm of 7568 (aging) was Non-traceable Author Statement

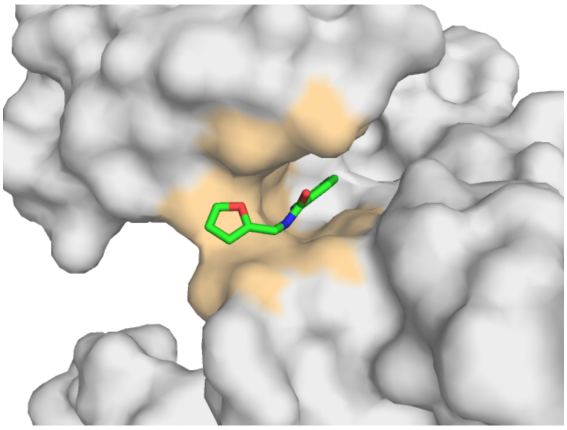

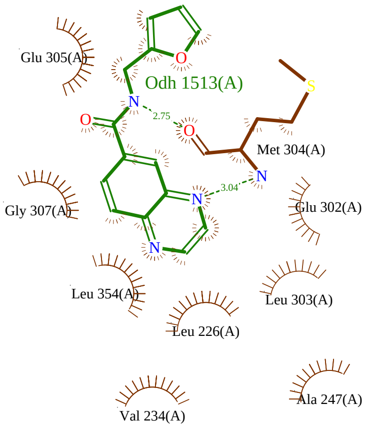

protein	amino acids contacts (binding site)								
PDB:4bdc:chainA:O96017	L	V	A	E	L	M	E	G	L
sp:O96017:CHK2_HUMAN	L	V	A	E	L	M	E	G	L
tr:Q9R019:Q9R019_RAT	L	V	A	E	L	M	E	G	L
sp:Q9Z265:CHK2_MOUSE	L	V	A	E	L	M	E	G	L
tr:Q543W6:Q543W6_MOUSE	L	V	A	E	L	M	E	G	L
tr:A4V0X0:A4V0X0_DROME	L	V	A	E	F	M	R	G	L
sp:O61267:LOK_DROME	L	V	A	E	F	M	R	G	L
tr:A4F324:A4F324_CAEEL	L	V	A	E	Y	V	G	G	L
sp:Q9U1Y5:CHK2_CAEEL	L	V	A	E	Y	V	G	G	L
sp:P22216:RAD53_YEAST	V	V	A	E	F	V	S	G	L

	whole protein		domain-based		contact-based	
protein	ident	simil	ident	simil	ident	simil
PDB:4bdc:chainA:O96017	1.0	1.0	1.0	1.0	1.0	1.0
sp:O96017:CHK2_HUMAN	1.0	1.0	1.0	1.0	1.0	1.0
tr:Q9R019:Q9R019_RAT	0.82	0.92	0.92	0.98	1.0	1.0
sp:Q9Z265:CHK2_MOUSE	0.82	0.92	0.92	0.98	1.0	1.0
tr:Q543W6:Q543W6_MOUSE	0.82	0.92	0.92	0.98	1.0	1.0
tr:A4V0X0:A4V0X0_DROME	0.31	0.64	0.43	0.81	0.78	0.91
sp:O61267:LOK_DROME	0.3	0.62	0.43	0.81	0.78	0.91
tr:A4F324:A4F324_CAEEL	0.28	0.63	0.36	0.75	0.67	0.82
sp:Q9U1Y5:CHK2_CAEEL	0.28	0.64	0.36	0.75	0.67	0.82
sp:P22216:RAD53_YEAST	0.16	0.4	0.38	0.73	0.56	0.82

lok (FBgn0019686) associated phenotypes

cell death defective, chemical resistant, conditional, decreased cell death, increased cell death, long lived, maternal effect, radiation sensitive

(Information from FlyBase)

lok (UniProt:O61267) annotation

Function: May have a role in germline establishment. (PubMed:9507063).
Subcellular location: Nucleus speckle(PubMed:9507063). Note=Speckled subnuclear compartment.
Tissue specificity: In stage 3 embryos, both isoforms are expressed in both somatic and pole cell nuclei. Expression in pole cell nuclei is sustained until stage 9 and weakly expressed after pole cell invagination into the abdominal cavity. (PubMed:9507063).
Developmental stage: Expressed both maternally and zygotically in adult females. Levels of the

long isoform remain fairly constant from ovaries to embryos, the levels of short isoform decrease dramatically. (PubMed:9507063).

(Information from UniProt)

chk-2 (UniProt:Q9U1Y5) annotation

Function: Serine/threonine-protein kinase which is required for checkpoint-mediated cell cycle arrest, activation of DNA repair and apoptosis in response to the presence of DNA double-strand breaks. May also negatively regulate cell cycle progression during unperturbed cell cycles. Phosphorylates and inhibits cdc25 phosphatase, preventing entry into mitosis. Required for nuclear reorganization and homologous chromosome pairing during meiotic prophase. (PubMed:11086161, PubMed:11158318).
Cofactor: Mg(2+)
Subcellular location: Nucleus (PubMed:11086161, PubMed:11158318).
Tissue specificity: Highly expressed in germline tissue. (PubMed:11086161).
Developmental stage: Low expression in embryos and L2 larvae, high expression in hermaphrodite adults. (PubMed:11086161).

(Information from UniProt)
